# Validating Cell Surface Proteases as Drug Targets for Cancer Therapy: What Do We Know, and Where Do We Go?

**DOI:** 10.3390/cancers14030624

**Published:** 2022-01-26

**Authors:** Emile Verhulst, Delphine Garnier, Ingrid De Meester, Brigitte Bauvois

**Affiliations:** 1 Laboratory of Medical Biochemistry, Department of Pharmaceutical Sciences, University of Antwerp, 2000 Antwerp, Belgium; Emile.Verhulst@uantwerpen.be (E.V.); ingrid.demeester@uantwerpen.be (I.D.M.); 2 Centre de Recherche des Cordeliers, Sorbonne Université, Inserm, Cell Death and Drug Resistance in Lymphoproliferative Disorders Team, F-75006 Paris, France; delphine.garnier@sorbonne-universite.fr

**Keywords:** cancer, protease, inhibitor, function, signalling, survival, drug resistance

## Abstract

**Simple Summary:**

Cell surface proteases (so-called ectoproteases) are associated with cancer, and their targeting may confer valuable options for the improvement of cancer treatment outcome. Over the past 20 years, the permanent development of a multitude of inhibitors against several ectoproteases (including DPP4, FAP, APN, ADAM17, MMP2, and MMP9) has made it into clinical evaluation in haematological and solid tumours. Among them, a few show some efficacy, albeit limited, to cure cancer in the near future. This Review summarizes the efforts thus far undertaken in the development of ectoprotease inhibitors and highlights new directions for targeting ectoproteases as an additional weapon in the fight against cancer.

**Abstract:**

Cell surface proteases (also known as ectoproteases) are transmembrane and membrane-bound enzymes involved in various physiological and pathological processes. Several members, most notably dipeptidyl peptidase 4 (DPP4/CD26) and its related family member fibroblast activation protein (FAP), aminopeptidase N (APN/CD13), a disintegrin and metalloprotease 17 (ADAM17/TACE), and matrix metalloproteinases (MMPs) MMP2 and MMP9, are often overexpressed in cancers and have been associated with tumour dysfunction. With multifaceted actions, these ectoproteases have been validated as therapeutic targets for cancer. Numerous inhibitors have been developed to target these enzymes, attempting to control their enzymatic activity. Even though clinical trials with these compounds did not show the expected results in most cases, the field of ectoprotease inhibitors is growing. This review summarizes the current knowledge on this subject and highlights the recent development of more effective and selective drugs targeting ectoproteases among which small molecular weight inhibitors, peptide conjugates, prodrugs, or monoclonal antibodies (mAbs) and derivatives. These promising avenues have the potential to deliver novel therapeutic strategies in the treatment of cancers.

## 1. Selection of Literature

The PubMed database (www.ncbi.nlm.nih.gov/pubmed, accessed on 19 December 2021) was employed to select papers for coverage in this review. The search terms “ectoprotease”, “cell surface protease”, “DPP4/CD26”, “FAP/Seprase”, “APN/CD13”, “ADAM17/TACE”, “MMP/gelatinase”, “drug”, “inhibitor”, “clinical trial”, “metabolism”, “tumour”, “microenvironment” were employed for this purpose. Although a few cited references were published prior to 2008, the majority of the cited references from the PubMed database were from 2008 to 2021.

## 2. Background and Introduction

**Figure 1:**
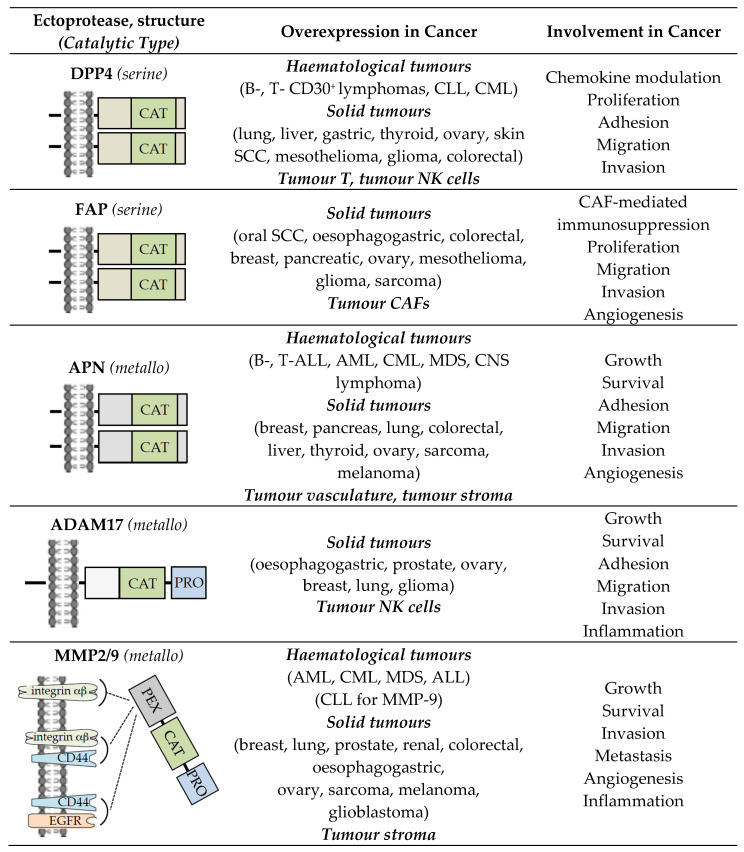
Structure, overexpression, and involvement of ectoproteases in cancer. The structures are simplified to show the domains of ectoproteases discussed in the text. DPP4, FAP, and APN are dimers of two noncovalently associated monomers. ALL, acute lymphoblastic leukaemia; AML, acute myeloid leukaemia; CAF, cancer-associated fibroblast; CAT, catalytic site; CLL, chronic lymphocytic leukaemia; CML, chronic myeloid leukaemia; CNS, central nervous system; EGFR, epidermal growth factor receptor; MDS, myelodysplastic syndrome; PEX, hemopexin domain; PRO, prodomain; SCC, squamous cell carcinoma. The catalytic type is mentioned according to The MEROPS database of proteolytic enzymes: https://www.ebi.ac.uk/merops/index.shtml (accessed on 16 December 2016).

Ectoproteases have been initially defined as transmembrane proteins with an active catalytic site exposed to the external surface of the membrane, and are represented by peptidases and ADAMs (a disintegrin and metalloprotease family) [1]. The roles of ectoproteases within neoplastic sites have been investigated actively. Attention has been peculiarly focused on dipeptidyl peptidase 4 (DPP4, CD26), fibroblast activation protein alpha (FAP, Seprase), aminopeptidase N (APN, CD13), and ADAM17 (also known as tumour necrosis factor-α-converting enzyme/TACE), whose deregulated expression in the tumour microenvironment (TME) is correlated with a malignant cancer phenotype (tumour cell growth, survival, metastasis, and tumour-associated angiogenesis) [2,3,4,5,6,7,8,9,10,11,12,13,14] (Figure 1). Through their enzymatic activities, DPP4, FAP, APN, and ADAM17 mediate the proteolysis of bioactive peptides and cytokines, components of the extracellular matrix, and transmembrane proteins (receptors and adhesion molecules) [8,10,11,12,13,15,16,17,18,19,20,21,22]. Ectoprotease interaction with their inhibitors or specific monoclonal antibodies (mAbs) has revealed that these ectoenzymes regulate intracellular key signalling pathways related to the modulation of major cell events (proliferation, survival, migration, angiogenesis) [11,12,13,18,20,22,23,24,25,26,27,28,29] (Figure 1). The enzymatic activity of DPP4, FAP, APN, and ADAM17, even if it contributes, is not essential for signal transduction. Indeed, these entities have a short intracytoplasmic tail, and inhibitor or mAb binding to the ectoenzyme initiates lateral membrane interactions with transmembrane proteins (including β1 integrins) for downstream signalling [14,18,28,30,31,32].

Today, the ectoproteases can also include proteases localized at the extracellular side of cell membranes. Two members of secreted matrix metalloproteinases (MMPs), i.e., MMP2 (gelatinase A) and MMP9 (gelatinase B) localize at the surface of tumour cells, and can be encompassed in the group of ectoenzymes [33] (Figure 1). As with DPP4, FAP, APN, and ADAM17, increased expression of MMP2/9 in TME is often associated with the development and progression of cancer [33,34,35], and both MMPs have the ability to cleave many different targets (extracellular matrix, cytokines, growth factors, chemokines, and cytokine/growth factor receptors) that in turn modulate key signalling pathways involved in cell growth, migration, invasion, and angiogenesis [33,36,37] (Figure 1). By binding to transmembrane proteins (αβ integrins, CD44, EGF receptor/EGFR), MMP2 and MMP9 directly trigger intracellular signalling pathways that control tumour cell events [33] (Figure 1). Thus, MMP2 and MMP9 can be considered as ectoproteases.

**Figure 2:**
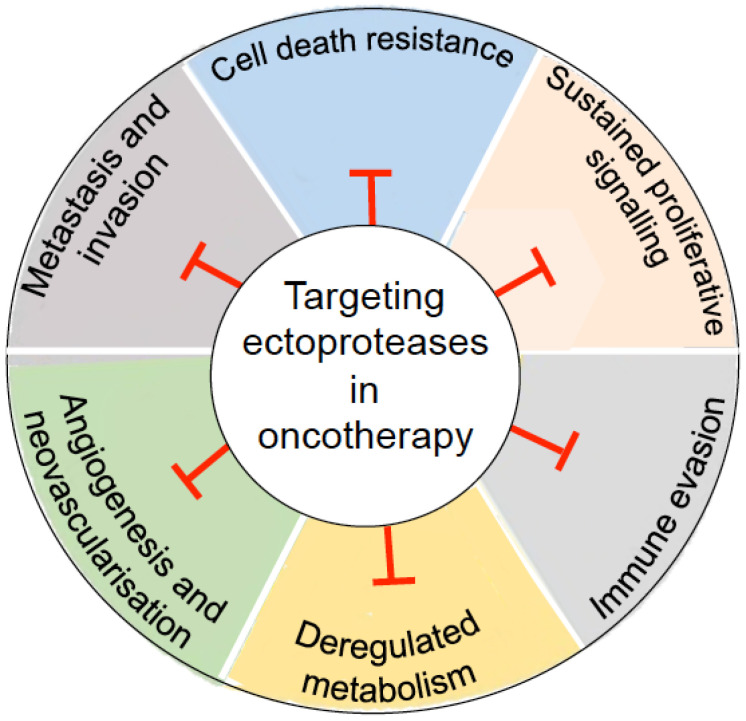
Cancer-associated ectoproteases as potential drug targets and implications in cancer therapy. Cancer is supported by a number of biologic hallmark capabilities that enable tumour development and progression. Ectoproteases participate in cancer by modulating a wide range of these processes. Drug candidates targeting ectoproteases may elicit antitumour effects (T) in a context-dependent manner.

The structures of these ectoenzymes, as well as their overexpression and roles in cancer, have been discussed in excellent reviews and will not be detailed here [4,11,12,13,18,20,23,26,27,33] (Figure 1). The detrimental roles of these ectoproteases expressed in TME has resulted in the design and development of a myriad of inhibitors, including small molecular weight molecules, synthetic peptide-based compounds, as well as mAbs. For an inhibitor to be clinically successful, it has to be selective for a given enzyme and it needs to accumulate in cancerous tissues without eliciting adverse effects. These properties make any program of drug discovery still difficult. Although most inhibitors developed so far against these ectoproteases showed no or limited anticancer activity in clinical trials, they are, however, crucial as starting points for designing improved ectoprotease targeting strategies for cancer therapy. In this Review, we critically summarize what is currently known about ectoprotease inhibitors and give an update on the newly developed drug candidates targeting these enzymes. Figure 2 summarizes the potential impact of drug candidates targeting tumour-associated ectoproteases on key signalling pathways and cellular processes of relevance to cancer that may bring a new dimension to the therapeutic approach of cancer.

## 3. DPP4/CD26

DPP4 is the best known member of the S9 family of serine proteases, besides DPP8, DPP9, FAP, and prolyl oligopeptidase [32]. DPP4 exhibits potent postproline dipeptidyl peptidase activity [32]. Among substrates of DPP4, chemokines are rapidly inactivated by DPP4, and the field of inhibitor research explored the possibility of therapeutic intervention to block chemokine processing by DPP4. The first DPP4 inhibitors turned out to be nonselective pan-DPP inhibitors inhibiting FAP, DPP8, and DPP9 [38,39,40]. Then, more selective active-site DPP4 inhibitors were designed and tested in patients with type 2 diabetes or cardiovascular diseases (for review in [41,42,43]). On the basis of their mode of action, DPP4 inhibitors are covalent (including vildagliptin and saxagliptin) and noncovalent (including sitagliptin, alogliptin, and linagliptin) inhibitors [44]. These inhibitors were approved by the U.S. Food and Drug Administration (FDA) and/or the European Medicines Agency for the treatment of type 2 diabetes [45]. Supported by their excellent safety profile [45], their effects were evaluated in several xenograft animal models of cancer [46,47,48,49,50,51]. For instance, linagliptin suppressed tumour growth in a xenograft mouse model of colorectal cancer [49]. Sitagliptin had a protective effect in a rat model of colon cancer, reducing the number of precancerous lesions in sitagliptin-treated animals [46], and it could overcome tyrosine kinase-inhibitor resistance in renal cell carcinoma spheroid cultures [50]. Sitagliptin treatment reduced melanoma growth in mice as a result of delayed chemokine processing [52], prolonged survival, and increased CD8^+^ T cell trafficking in a syngeneic ovarian cancer mouse model, highlighting the importance of DPP4 in the regulation of the immune landscape [51]. Two large meta-analyses of clinical trials studying the impact of alogliptin, linagliptin, saxagliptin, sitagliptin, and vildagliptin in the survival of patients with lung or colorectal cancer between 2007 and 2013, and prostate, pancreas, or breast cancer between 2007 and 2015, showed that DPP4 inhibition improved survival in patients with lung, prostate, and colorectal cancer [53,54]. At the same time, a thorough follow-up of long-term DPP4 inhibition remains a valuable preventive measure, as the influence of DPP4 on cancer biology remains complex [55]. Linagliptin and sitagliptin are now tested in phase I/II clinical trials to treat oesophagogastric and non-small cell lung cancer (Table 1) [56]. These promising findings provide rationale to figure out the potential of DPP4 inhibitors in combination with checkpoint immunotherapy or chemotherapy. It has to be pointed that DPP4 can modulate immune effects in a nonenzymatic manner by interacting with other transmembrane proteins [57] or extracellular adenosine deaminase (ADA) [58]. ADA catalyzes the irreversible deamination of adenosine and 2′ deoxyadenosine to inosine and 2′ deoxyinosine, respectively. Adenosine is highly immunosuppressive for most of immune cells. Adenosine interferes with TCR signalling by binding to the adenosine receptor 2a expressed on effector T cells [59]. Exogenous ADA bound to T cell surface DPP4 has the ability to metabolize immunosuppressive adenosine, which ensures T cell proliferation [60] and to produce a CD3-dependent costimulatory downstream signal for T cell activation [60,61]. Interaction of the ADA–DPP4 complex on T cells with an ADA-anchoring protein on dendritic cells results in the production of inflammatory cytokines by the latter [62]. These effects highlight the importance of adenosine and ADA–DPP4 pathways in the modulation of T cell immune responses. Although caution has to be taken when targeting DPP4 for the treatment of cancer, DPP4 inhibitors tested in clinic so far have proved to be safe therapeutics [45], and they deserve further exploration in combination treatments of cancers with an unmet clinical need.

Apart from catalytic site inhibitors, DPP4 has been clinically targeted by mAbs. The humanized antibody YS110 was initially developed as a targeted therapy against CD26^+^ malignancies [63]. It exerts a very effective antitumour effect in renal cancer, malignant mesothelioma, and malignant lymphoma [63,64,65]. In phase I/II trials, YS110 led to prolonged stable disease and moderate side effects in patients with malignant pleural mesothelioma (MPM) and renal cell carcinoma, suggesting its potential as a better tolerated and more effective therapy in these cancers [66]. In a recent phase I/II study evaluating YS110 in patients with advanced MPM, it was generally well tolerated and showed some efficacy as a salvage therapy in difficult-to-treat patients (Table 1) [67,68]. The YS110-based antibody–drug conjugate Y-TR1 containing triptolide (TR-1, Nrf2 inhibitor) is in the preclinical phase [69,70]. The clinical utility of these mAbs deserves future attention.

One recent immunotherapy involves the use of chimeric antigen receptor (CAR)-T cells, which relies on cytotoxic T cells being modified to express an artifical receptor targeting cancer-specific surface antigens, and is infused into the patients, where they recognize and eliminate the tumour [71,72]. CARs, as transmembrane proteins, bind their cognate targets through their extracellular domain and bear signal transduction capacities that trigger the cytotoxic functions of host effector T cells [71,72]. DPP4 targeting CAR-T cells were developed to target DPP4^+^ leukemic stem cells in chronic myeloid leukaemia (CML) [73,74,75,76]. To broaden the success of CAR-T cell treatment for CML, Zhou et al. attempted to construct second-generation DPP4 targeting CAR-T cells utilizing 4-1BB (CD137) as costimulatory domain to target leukaemia stem cells [76,77]. Although limiting growth tumour progression in a mouse model, anti-DPP4-4-1BB CAR-T cells exhibited self-antigen-driven fratricide, indicating that a more optimized design or alternative target is needed [76,77].

## 4. FAP

FAP features a unique dual dipeptidyl peptidase and endopeptidase activity, which distinguishes it from DPP4 and other members of the S9 family [99,100]. The first inhibitors Glu-boroPro (PT-630) and Val-boroPro (PT-100, talabostat^®^) target not only FAP, but also DPP4 and/or DPP8/9 (reviewed in [10,101]). They had no effect on tumour cell growth in vitro or in immunodeficient mice, suggesting that these inhibitors targeted the immune system [57,102]. The involvement of the immune system in the antitumour activity of Val-boroPro was confirmed in mice depleted with CD4^+^ and/or CD8^+^ T cells [103]. The parallel inhibition of DPP4 and/or DPP8/9 by Val-boroPro complicated the proper interpretation of the results [52,104]. An adaptive phase I trial was developed to define the optimal dose of Val-BoroPro in children with relapsed or refractory solid tumours [105]. Val-BoroPro, in combination with cisplatin or docetaxel, reached phase II clinical trials in lung cancer, metastatic colorectal cancer, and melanoma, however clinical evaluation was terminated because Val-BoroPro did not improve the clinical activity of conventional drugs (possibly due to poor patient selection and poor trial design) [106,107,108,109]. Instead, recent research supports DPP9 as the preferred target of Val-boroPro [52,104]. Val-boroPro, ‘’redefined’’ as DPP8/9 inhibitor BXCL701, is currently being evaluated in combination with checkpoint inhibitors in metastatic castration-resistant prostate cancer [110,111,112]. ARI-4175, a second-generation pan-DPP inhibitor following Val-boroPro, mediates tumour regression through immune-mediated mechanisms [113]. ARI-4175 treatment induces the marked regression of well-established lung tumours, both as a single agent and as an adjuvant to dendritic cell therapy and adoptive cellular therapy [114], and significantly lowers the total number of macroscopic liver nodules in mouse models of lung cancer [115]. Whether pan-DPP inhibitors remain suitable candidates for treating certain cancers warrant further clinical investigation.

Insights into the structural characteristics of the active site and substrate preferences of FAP led to the development of FAP-specific inhibitors [116,117]. These include (4-quinolinoyl)glycyl-2-cyanopyrrolidine-based inhibitors [116,117]. One selective FAP inhibitor with low nanomolar potency is UAMC-1110 [118]. UAMC-1110 did not affect tumour growth, nor did it enhance the effect of radiotherapy in a mouse model of pancreatic cancer [119]. However, MIP-1232, a UAMC-1110-based probe radiolabeled with I^125^, appeared to be highly correlated with tumour tissue imaging [120]. Based on their selective and strong binding to FAP, a multitude of radiolabeled FAP inhibitors (FAPI) have been generated and are now widely used in experimental tumour diagnosis (reviewed in [10,14]). Several FAPI tracers recently entered the clinical phase for pan-tumour imaging, diagnosis, and staging examinations (Table S1) [121]. To turn the FAPI radiopharmaceuticals into theranostics, the linker region was modified to obtain improved tumour retention and to allow the use of radionuclides suited for therapy [122]. Probes such as ^177^Lu-FAP-2286, ^64^Cu-FAPI-04, ^225^Ac-FAPI-04, and RPS-309 were developed as theranostics [123]. First-in-human results of ^177^Lu-FAP-2286 demonstrate a long retention time in diverse solid tumours (advanced adenocarcinomas of pancreas, breast, rectum, and ovary) and acceptable side effects (Table 1) [78]. Prospective clinical studies are warranted.

An alternative FAP-directed cancer therapy consists of FAP-mediated activation of prodrugs. The specific gly-pro-directed proteolytic activity of FAP has been exploited in the development of several FAP-activatable prodrugs [124]. Upon cleavage by FAP, the nontoxic prodrug is activated to a potent toxin that kills both FAP^+^ and neighbouring FAP^−^ cells in the tumour [124]. The ERGETGP-S12ADT-activated prodrug generated by coupling a FAP cleavable peptide to a thapsigargin analogue inhibits tumour growth in human breast and prostate cancer xenograft models with no associated toxicity [125,126]. Other FAP-targeted prodrugs include compounds based on promelittin [127], emetine [128], arenobufagin [129], desacetyl-vinblastine [130], or doxorubicin [131]. Unexpectedly, these promising prodrugs encountered a roadblock in preclinic.

Another approach aims at the development of mAbs for the selective inhibition of FAP. The first of them, mAb F19, exhibited a tight selectivity for FAP^+^ fibroblasts in tumour tissues [132]. Thereafter, several humanized versions of F19 were developed with limited or no antitumour activity (reviewed in [10]). Among them, sibrotuzumab was tested in phase I/II clinical trials in patients with metastatic colorectal cancer, albeit without therapeutic response, and some patients developed antihuman Abs [133,134]. Thereafter, FAP Abs have been conjugated with toxins, radioisotopes, immunomodulatory cytokines, or costimulatory molecules (reviewed in [10,14]). Based on the role of the costimulatory receptor 4-1BB (CD137, TNFRSF9) in sustaining effective T cell immune responses, a bispecific FAP antibody-fusion protein combining a trimeric 4-1BB ligand (4-1BBL) and a Fab moiety recognizing fibroblast FAP was developed [135]. Simultaneous binding of anti-FAP to CAFs and 4-1BBL to T cells resulted in the clustering and activation of T and NK cells at the tumour site, thereby leading to potent antitumour activity in mice xenograft models [135,136]. Anti-FAP-4-1BBL is currently tested in the clinic as single agent or in combination with cibisatamab (CEA/CD3 bispecific mAb) or atezolizumab (ATZ) (anti-PD-L1) (Table 1) [79]. First-in-human results confirmed tumour-specific uptake and a favourable safety profile, which supports further clinical exploration [79,80] (Table 1). A bispecific FAP-CD40 mAb (RO7300490) applies a similar principle to act as costimulatory signal for antigen-presenting cell (APC) activation, leading to enhanced T cell priming and tumour regression in mice xenograft models without clear signs of toxicity [137]. The FAP-directed CD40 agonist has progressed to clinical trial phase I as a single agent or in combination with ATZ (Table 1). The tetravalent bispecific mAb RG7386 (RO874813, a FAP mAb coupled with death receptor 5 (DR5) agonist), which binds FAP^+^ fibroblasts and DR5^+^ tumour cells, induced tumour regression in a colorectal cancer mouse model [138]. Although a phase I clinical trial with RG7386 demonstrated a favourable safety profile in patients with multiple solid tumour types and antitumour activity in a patient with non-small cell lung cancer [139,140], its development has been discontinued. A FAP mAb combined with variant interleukin-2 (IL-2v) (simlukafusp α, SIM) has been developed, with IL2v binding IL2-Rβγ but not IL2-Rα on T cells [141]. As a result, SIM strongly activates NK and CD4^+^/CD8^+^ T cells, but not Tregs, and therefore may augment activity of PD-(L)1 inhibitors [141]. SIM entered the clinic as monotherapy, in combination with ATZ [82], trastuzumab (anti-HER2) or cetuximab (anti-EGFR) [79], ATZ ± bevacizumab (anti-VEGF) [81,82], or pembrolizumab (anti-PD-1) (Table 1). In phase I studies, SIM was associated with an acceptable safety profile in patients with advanced solid tumours (Table 1) [79,81]. In a phase II study, SIM, in combination with ATZ, confirmed the safety profile in patients with cervical squamous cell carcinoma (Table 1), supporting the further exploration of SIM with checkpoint inhibition in this patient population [82].

Oncolytic group B adenoviruses have been previously optimized for selective tumour cell infection and stability in blood [142,143]. An interesting FAP-targeting strategy concerns the recent development of NG-641, a modified variant of the adenovirus enadenotucirev that encodes a bispecific single-chain diabody (bispecific T cell engager/BiTE) to simultaneously bind FAP^+^ CAFs and CD3^+^ T cells [144]. NG-641 also encodes the transgenes CXCL9, CXCL10, and IFNα to recruit T cells and enhance the overall immune response and cancer cell killing [145,146]. In addition to the infection of tumour cells with the oncolytic virus NG-641, the encoded BiTE should lead to potent T cell activation and CAF death [144]. This approach yields a multimodal treatment strategy within a single therapeutic agent. The study of the safety and tolerability of NG-641 in patients with metastatic or advanced epithelial tumours started in 2020 (Table 1).

As mentioned above for DPP4, several anti-FAP CAR-T models based on MO36 and F19 mAbs were constructed (reviewed in [10,14]). A blockade of tumour growth by anti-FAP CAR-T cells was validated in human lung cancer xenografts and syngeneic murine pancreatic cancers [147]. The high efficacy of combining anti-FAP CAR-T cells with other immunotherapies (e.g., checkpoint inhibition) was proven by Gulati et al., who achieved transiently stable disease in a humanized fibrosarcoma mouse model treated with anti-FAP (F19 mAb) CAR-T cells in combination with a PD-1-blocking mAb [148]. In a phase I clinical trial, intra-pleural administration of autologous anti-FAP CAR-T cells to three patients with malignant pleural mesothelioma was well tolerated without any evidence of treatment-related toxicity (Table 1) [84]. A Nectin4/FAP-targeted fourth-generation CAR-T cells (expressing IL7 and CCL19, or IL12) therapy is currently being tested to treat Nectin4^+^ advanced malignant solid tumours (Table 1).

## 5. APN/CD13

APN/CD13, as a member of the M1 metallopeptidase family, harbours a Zn^2+^-binding motif in its active site [149,150]. The first described natural or synthetic APN inhibitors exhibited a zinc binding group such as hydroxamate, carboxylate, sulfhydryl, sulfonamide, and derivatives of phosphoric acid in their moieties [151,152,153,154]. However, they lacked tight specificity by inhibiting other metalloproteases [151]. The best-known example is bestatin (Ubenimex^®^), which entered the clinic in the 1990s for the purpose of treating patients with haematological and solid tumours including acute and chronic leukaemias, lymphomas, melanoma, lung, bladder, and stomach carcinomas [151,152,153]. Although bestatin showed few adverse effects, its survival benefit did not appear significant. Most studies were small or had few recorded events, and, in several cases, the positive results were restricted to subgroups’ analyses (reviewed in [8]). More recently, in an early nonrandomised study, bestatin was shown to reduce the polyp number in patients with colorectal cancer [155]. Thereafter, many new APN inhibitors have been designed and synthesized, including derivatives of 3-amino-2-hydroxy-4-phenyl butanoic acid, chloramphenicol amine, 3-phenylpropane-1, 2-diamine, L-lysine, L-arginine, 1, 3, 4-thiadiazole, N-cinnamoyl-L aspartic acid, and cyclic-imide moieties, most of them being still in the (pre)clinical stage of development [153,156]. The cyclopentyl ester CHR-2797 (tosedostat) [157] was investigated both as a monotherapy and in combination with other drugs [8]. In phase I/II trials, CHR-2797 was found safe and effective in relapsed and refractory acute myeloid leukaemia (AML) [158,159,160]. When combined with low dose cytarabine, decitabine, or azacitidine, CHR-2797 was not associated with major toxic effects in a small cohort of patients with AML [161,162] or myelodysplastic syndrome (MDS) [86,161] (Table 1). However, phase II randomised studies with a large AML sample size demonstrated that the addition of CHR-2797 to standard chemotherapy negatively affects the therapeutic outcome of AML patients due to more infection-related deaths [163,164]. With regard to solid tumours, a phase I study of CHR-2797 monotherapy demonstrated tolerability and preliminary efficacy in a subset of patients with advanced renal, colorectal, lung, prostate, breast, and pancreatic tumours [165]. A recent phase Ib/II study combining CHR-2797 with capecitabine in patients with advanced pancreatic adenocarcinoma displayed tolerable toxicity in a cohort of 16 patients; however, due to insufficient funding and drug supply from manufacturer, the clinical study was terminated (Table 1) [85]. These observations of CHR-2797 in solid tumours warrant further clinical investigation.

Based on its ability to bind to the Asn-Gly-Arg (NGR) motif, APN has proven to be a key for targeted delivery of chemotherapeutic drugs to APN^+^ tumour cells and APN^+^ tumour-associated endothelium [8,166]. Interestingly, the NGR motif binds to APN isoforms in tumoural tissues, but not to normal APN-rich tissues [8]. A large variety of molecules have been coupled to the NGR motif (which can be flanked by two cysteine moieties in a circular CNGRC peptide), including cytotoxic agents (doxorubicin, 5′ fluoro-2′-deoxyuridine, 5-fluorouracil, lidamycin), cytokines (TNF-α, IFN-γ), and anti-angiogenic peptides (endostatin, truncated tissue factor/tTF, D(KLAKLAK)_2_) [8,166,167,168,169,170]. The NGR-coupled drugs showed antitumour activity in vitro and in preclinical models of haematological and solid tumours [168,169,170,171]. As mentioned above for FAP, preclinical research developed APN-targeted molecular imaging probes for the noninvasive detection of angiogenesis in vivo; among them, a dimeric NGR-containing peptide conjugated with a chelator, and radiolabeled with ^64^Cu, was shown to be a suitable radioprobe [168,172]. For 10 years, NGR-TNF-α has been tested (both as a single agent and in combination with chemotherapy) in several phase I/II clinical trials in patients with advanced solid tumours (including melanoma, small cell lung cancer, colon, liver, and ovarian carcinomas) (reviewed in [8,167,169,173]). In phase II/III studies, NGR-TNF-α showed manageable toxicity and promising activity in primary central nervous system lymphoma [87], small cell lung cancer [88], and malignant pleural mesothelioma [89] (Table 1). Recently, a prospective phase I trial with the antiangiogenic drug NGR-tTF was initiated for patients with recurrent or refractory malignant tumours and lymphomas (Table 1) [90]. These innovative approaches deserve to be pursued.

Another way of targeting APN is mAbs. APN mAbs (WM15, MY7, and SJ1D1 epitopes) were able to induce in vitro the death of primary AML cells and liver cancer stem cells (CSCs), and to slow tumour growth in a xenograft murine model of liver carcinoma [24,174]. APN mAb TEA1/8 conjugated to the marine compound PM050489 (which binds tubulin and thus impairs microtubule dynamics) exhibited antitumour activity in APN^+^-fibrosarcoma xenograft murine models [175]. A bispecific Ab generated by combining a CD3 Fab (OKT3) and an APN Fab (MY7) reacts with both CD3^+^ T cells and APN^+^ AML cells, leading to the elimination of AML cells by peripheral blood mononuclear cells [176]. Using a modified approach of CAR-T cells, He et al. constructed a switchable CAR-T system based on an APN mAb (Nb157) which eliminates primary AML cells in vitro and in an AML mouse model [177]. As a whole, these approaches suggest that mAbs may be a therapeutic option in the treatment of APN^+^ tumours.

An alternative therapeutic strategy exploited the proteolytic activity of APN for the activation of prodrugs. In peculiar, the promising activity of the alkylating prodrug melflufen (J1) is related to the ability of tumour APN to directly turn melflufen (dipeptide consisting of melphalan and *p*-fluoro-L-phenylalanine) into an active cytotoxic drug, melphalan (Table 1) [178]. In a phase I/IIa clinical study of solid tumours, clinical activity was suggested in ovarian cancer, but only modest activity in refractory non-small cell lung cancer [178]. In phase I/II studies (HORIZON and ANCHOR), melflufen plus dexamethasone has demonstrated encouraging clinical activity and a manageable safety profile in heavily pretreated patients with relapsed/refractory multiple myeloma (RRMM) (Table 1) [91,92]. OCEAN, a randomised phase III study, evaluated the efficacy and safety of melflufen + dexamethasone versus pomalidomide + dexamethasone (Table 1) [92,93]. Melflufen plus dexamethasone showed clinically meaningful efficacy and a manageable safety profile in patients with heavily pretreated RRMM, including those with triple class refractory and extramedullary disease (Table 1) [92].

## 6. ADAM17/TACE

As with APN, the active site of the metalloprotease ADAM17 is dependent on Zn^2+^ for its catalytic activity and can bind molecules that have a zinc binding group (such as hydroxamate, sulfonamide, tartrate, and hydantoin) in their structures. In this context, a large variety of potential low molecular weight ADAM17 inhibitors have been developed over the last 20 years [21,179]. Among them, DPC333 (BMS-561392) [180], PF-5480090 (TMI-002, WAY-18022) [181], TMI-005 (apratastat) [182], INCB3619 [183,184], and INCB7839 (aderbasib) [185] have entered phase I/II clinical trials for the management of inflammatory diseases and solid tumours (including breast cancer and non-small cell lung cancer); however, due to side effects and a lack of selectivity, they had to be withdrawn later on [13,19,20,167,179]. Indeed, these compounds also inhibit MMP8/13 (for TMI-005 and PF-5480090) or ADAM10 (for INCB3619 and INCB7839) [19,179]. In 2014, INCB7839 again entered a phase I/II clinical trial, to be used along with rituximab (anti-CD20) as consolidation therapy after an autologous haematopoietic cell transplant for patients with diffuse large B cell non-Hodgkin lymphoma (Table 1) (reviewed in [13,179]). The short-term results suggest its applicability as a relapse-preventing therapy [94].

Several ADAM17 mAbs including D1 (A12) [186,187], A9 (B8) [188], A300E, and related conjugates [189,190,191], as well as MEDI3622 [192], have been developed. D1 mAb binds to the ectodomain outside the catalytic site of ADAM17 [187]. A9 mAb binds the ectodomain of ADAM17 and directly causes a conformational change involving the ADAM17 catalytic site [188]. Both D1 and A9 mAbs exhibit antitumour effects in various cancer models including breast, head, neck, and pancreatic cancers [193,194,195]. The A300E mAb was initially developed against the disintegrin–EGF-like domain of human ADAM17 [189]; when conjugated to doxorubicin or Pseudomonas exotoxin, it induces in vitro death of breast cancer cells in an ADAM17-dependent manner [191]. An A300E-specific scFv (single-chain variable fragment) was coupled to a CD3-specific scFv to generate a bispecific T cell engager antibody (A300E-BiTE) which is capable to induce in vitro the T cell-mediated lysis of prostate cancer cells [190]. The mAb MEDI3622 recognizes a unique hairpin loop in the ectodomain of the enzyme (and absent in other ADAMs and MMPs) [186]. By blocking the ADAM17-mediated shedding of HB-EGF (ligand of the EGF receptor/EGFR), MEDI3622 exhibits antitumour activity in xenograft models of EGFR-dependent colorectal and oesophageal cancer [192,196,197]. The promising preclinical efficacy seen with these ADAM17 mAbs supports their further clinical investigation.

The propeptide domain of metalloenzymes including ADAM17 and MMP2/9 is characterized by an amino acid sequence known as “cysteine switch”, in which the cysteine residue contains a sulfhydryl group coordinated to the catalytic divalent zinc ion to suppress the catalytic activity of the MMP [198]. The cleavage of the prodomain leads to the active form of MMPs [198]. Taking this into account, a recombinant prodomain peptide of ADAM17 was synthesized and shown to be an effective and highly specific inhibitor of ADAM17 activity in sepsis and inflammation models [199] and a murine kidney fibrosis model [200]. Recently, Soto-Gomez et al. [201] have developed a bispecific fusion protein construct (E0-GS-TPD) consisting of the inhibitory prodomain peptide of ADAM17 fused to an EGFR-targeting design ankyrin repeat protein, which inhibits the proliferation of lung cancer cells [201]. The use of such specific proteins could be an innovative strategy for the treatment of EGFR-dependent cancers.

## 7. MMP2 and MMP9

Numerous inhibitors of the catalytic activity of MMPs have been developed. Most of them bind to the Zn^2+^ ion and the substrate binding pocket and aim to target MMP2 and/or MMP9 [34,198,202,203,204,205,206]. These inhibitors include the well-known BB-94 (batimastat), BB-2516 (marimastat), and BAY12-9566 (tanomastat) [34,202,203]. Unfortunately, in phase III clinical trials, these compounds failed as drugs for the treatment of different types of solid tumours, due to significant dose-limiting musculoskeletal toxicity and/or lack of selectivity for individual MMPs [34,204,205,207]. Later on, more selective MMP2 or MMP9 inhibitors were developed. They included a series of aryl-sulfonamide, aryl-sulfonide, aryl sulfonyl based-glutamine, aryl carboxamide-based isoglutamine, and biphenyl-substituted lysine derivatives with affinities in the low nanomolar range. Whether these compounds are effective in clinic has not been shown to date [207,208,209,210].

Therefore, the question remains as to whether the therapeutic targeting of MMP2 and MMP9 is feasible. In light of the current insights in the nonproteolytic (i.e., outside-in signalling) roles of (pro)MMP2 and (pro)MMP9 [33], the enzyme inhibitor approach may no longer be sufficient because it does not affect the interactions of MMP2 and MMP9 with cell surface proteins and consequent signalling. Earlier studies showed that the interaction between (pro)MMP9 and its docking receptors (αβ integrins and CD44) requires an intact MMP9 hemopexin domain (PEX) [211,212,213]. In contrast to the highly conserved MMP catalytic domain, the PEXs are unique to each MMP family member. Taking this into account, Bjorklund et al. [211], in a pioneering study, developed an inhibitory peptide (ADGACIL WMDDGWCGAAG) that binds selectively to the MMP9 PEX domain and prevents PEX from binding to αvβ5 integrin; consequently, this peptide prevents tumour xenograft growth in vivo [211]. Thereafter, two more potent MMP9 PEX inhibitors have been described [213,214]. They prevent the association of MMP9 with its receptors (α4β1 integrin/CD44), resulting in the blocking of a downstream signalling pathway required for MMP9-mediated tumour cell migration in vitro and the inhibition of tumour metastasis in xenograft mouse models of lung cancer [213,214]. Scannevin et al. designed and synthesized a small molecule known as JNJ0966 [N-(2-((2-methoxyphenyl)amino)-4′-methyl-[4,5′-bithiazol]-2′-yl) acetamide] which prevents the conversion of proMMP9 (latent form) into the catalytically active enzyme [215]. JNJ0966 penetrates the blood–brain barrier and reduces severity in a mouse experimental autoimmune encephalomyelitis model [215]. Although these new approaches hold promise due to enhanced selectivity toward MMP9, the clinical utility of these compounds as therapeutic agents in cancer remains to be investigated.

Newly designed inhibitors include function-blocking MMP9 mAbs [202,206,216,217]. The first developed mAbs, REGA-3G12 [218,219] and CLAY-001 [220], inhibited the enzymatic activity of human MMP9. Their therapeutic efficacy was demonstrated ex vivo in autoimmune skin cells for REGA-3G12 [218] and in a xenograft model of intestinal fibrosis for CLAY-001 [220]. Thereafter, two allosteric mouse MMP9 mAbs, AB0041 and AB0046, were shown to inhibit tumour growth and metastasis in a xenograft model of colorectal carcinoma [221,222]. A humanized version of AB0041, GS-5745 (andecaliximab) inhibits MMP9 through two mechanisms: binding to proMMP9 prevents MMP9 activation, whereas binding to active MMP9 allosterically inhibits enzymatic activity [221,223]. Phase Ib and II/III trials in patients diagnosed with ulcerative colitis [224,225] and a phase Ib trial on rheumatoid arthritis patients [226] demonstrated that GS-5745 was safe and well tolerated. Since then, clinical trials evaluating GS-5745 in combination with anticancer drugs have been initiated. Phase I studies of GS-5745 as a monotherapy and in combination with chemotherapy in patients with advanced solid tumours (pancreatic, non-small cell lung, oesophagogastric, colorectal, and breast cancers) demonstrated encouraging antitumour activity without added toxicity (Table 1) [95,96,97]. In a phase III study, the addition of GS-5745 to mFOLFOX6 (a combination chemotherapy that includes oxaliplatin, leucovorin, and 5-fluorouracil) in first-line therapy did not improve overall survival in unselected patients with untreated EGFR2-negative oesophagogastric adenocarcinoma (Table 1) [98]. The efficacy of GS-5745-based MMP9 inhibition, either as a monotherapy or in combination with chemotherapy, remains to be tested in other tumours.

Remarkably, the progress in the design of specific MMP2 inhibitors has been slower than for MMP9 [202,205]. A macromolecular inhibitor designed to interact with both the active site and the PEX of MMP2, linking a MMP2 selective inhibitory peptide (APP-IP, a β-amyloid precursor protein) to the N-terminus of tissue inhibitor of metalloproteinases-2 (TIMP2), could inhibit fibrosarcoma cell migration [227]. With regard to proMMP2 targeting, Sarkar et al. [228] recently developed a cyclic peptide (cy(WPHPY)) which binds to proMMP2 and disrupts the interaction between proMMP2 and TIMP-2, thereby preventing TIMP2-mediated proMMP2 activation and inhibiting cell invasion of human melanoma cells [228]. These MMP2 inhibitors have yet to further prove their clinical efficacy.

## 8. New Avenues for Ectoproteases in the Context of Cancer Therapy

Recent observations revealed new hallmarks of cancer-associated ectoproteases. This section highlights (i) the relevance of ectoproteases in cancer stem cells (CSCs) and tumour-associated extracellular vesicles (EVs) and (ii) the interplay between ectoproteases and tumour metabolism.

TME is composed of an extracellular matrix, circulating factors (cytokines, growth factors, chemokines…), and several cell types, including differentiated cancer cells, CSCs, mesenchymal stem cells, cancer-associated fibroblasts (CAFs), endothelial cells, and immune cells [229,230,231,232]. All these components contribute to tumour growth, metastasis, angiogenesis, resistance to drugs, and escape from immune surveillance [229,230,231,233]. Such TME heterogeneity hampers effective cancer management in clinical practice, and novel therapeutic strategies are needed [229]. As discussed in this Review, the inhibitors developed so far effectively inhibit the activity and/or function of ectoproteases originating from mature tumour cells, endothelial cells, as well as CAFs and immune cells. Recent studies have indicated that CSCs express DPP4, APN, ADAM17, and MMP2/9 [12,73,234,235,236,237,238,239,240]. The anti-DPP4 14D10 (YS110 precursor) elicits significant efficacy against MM by impairing in vitro mature MM cells as well as the stem cell side population in murine xenograft models [241]. Liver APN^+^ CSCs are killed in vitro by NGR-lidamycin [170]. Meanwhile, bestatin and anti-APN WM15 enhance in vitro the hypersensitivity of liver CSCs to 5-fluorouracil treatment [174]. By inhibiting ADAM17 activity in liver CSCs, the broad-spectrum inhibitor of metalloenzymes TAPI-2 blocks Notch activation, responsible for a more aggressive phenotype [238]. CSCs derived from lung adenocarcinoma display high levels of MMP2, which contribute to highly invasive and migratory cell capabilities [242]. These few examples emphasize the relevance of ectoprotease inhibitors in targeting and eliminating CSCs. Moreover, a growing number of studies have revealed the presence of ectoproteases in EVs derived from TME. EVs are small cell-derived membrane vesicles, produced either through the endosomal pathway, giving rise to exosomes, or after budding of plasma membrane, resulting in microvesicles [243,244,245,246]. EVs obtained from the serum of cancer patients holds promise as diagnostic and prognostic parameters [229,247,248]. EVs influence tumour growth, metastasis, epithelial-to-mesenchymal transition, and drug resistance [229,247,248,249]. Exosomes transfer various molecules from tumour cells to immune cells and neovascular cells, contributing to the escape from immune surveillance and increased angiogenesis, respectively [231,245,246]. DPP4, APN, ADAM17, and MMP2/9 were recently described in EVs derived from solid and haematological tumours [242,250,251,252,253,254,255,256,257]. For instance, DPP4^+^ exosomes in AML patients’ plasma suppress the proliferation of normal haematopoietic progenitor cells, and, diprotin A, by inhibiting DPP4 activity, reverses the effects of exosome-mediated myelosuppression [251]. The inhibition by sitagliptin and vildagliptin of exosomal DPP4 derived from 5-fluorouracil-resistant colon cancer cells suppresses tumour growth and angiogenesis in vivo [258]. EVs from myeloid tumours (including AML, CML, MDS, and myeloproliferative neoplasms) contain high levels of APN [250]. Patients with metastatic colorectal cancer (CRC) express high serum levels of exosome-derived ADAM17 which contribute to metastasis formation by cleaving E-cadherin junctions in the subsequent premetastatic niche [242]. Moreover, the interaction between the integrin α5β1 on CRC cells and its ligand ADAM17 on exosomes mediates the uptake of exosomes by cancer recipient cells, which can bear relevance during the peritoneal dissemination of CRC [259]. Enhanced expression of MMP2/9 in exosomes from prostate cancer is correlated to tumour progression [260]. MMP9^+^DPP4^+^ exosomes derived from glioma are potent inducers of angiogenesis ex vivo through the phenotypic modulation of endothelial cells [261]. In addition, exosomes carry genetic materials, including DNA, mRNA, miRNA, long noncoding (lnc) RNA, and circular (cir) RNA [262,263]. Deregulated miRNA, lncRNA, and cirRNA enhance mRNA expression in human cancers [262,263]. Exosomal miRNAs (miR-100-5p and miR-21-5p) derived from prostate CSCs increase MMP2/9 expression and enhance the MMP-mediated migration of tumour cells, contributing to local invasion and premetastatic niche formation [264]. Exosomes released by liver and lung tumour cell lines can deliver cir-MMP2 and lnc-MMP2 RNAs to recipient tumour cells, respectively, leading to increased MMP2 expression and an MMP2-mediated invasion of tumour cells [265,266]. As a whole, these observations strongly suggest the importance of ectoproteases in the behaviour of CSCs and EVs. An aera in development concerns the therapeutic targeting of CSCs [230,231,232] and EVs [245,247,248]. Thus, ectoproteases originating from CSCs and EVs represent good target candidates, as their inhibition may open a window in designing new, effective strategies to eliminate CSCs and EVs derived from TME.

The second insight that deserves attention concerns the increasing evidence of the interplay between ectoproteases and tumour cell metabolism. Metabolic reprogramming is a common phenomenon in haematological and solid tumours. In TME, different cell subpopulations reprogram their metabolism to survive, proliferate, metastasize, and develop resistance to cancer therapies [267,268]. Alterations of metabolic pathways include upregulated glycolysis, glutaminolysis, fatty acid catabolism, and low or impaired oxidative phosphorylation [267,269]. The production and removal of reactive oxygen species (ROS) is involved in glucose and glutamine metabolism, and vice versa [267,269,270]. There is increasing evidence for a bidirectional link between ectoproteases and tumour metabolism. For instance, increased expression of APN from liver CSCs promotes cell survival by limiting the increase in ROS [271]. APN-mediated downregulation of ROS levels results in the inactivation of the MAPK signalling pathway and increased expression of multidrug resistance-associated proteins (including ABC transporters) [272]. Activation of tyrosine metabolism by APN in liver CSCs contributes to stem cell maintenance, thus leading to tumour relapse [273]. Similarly, MMP9 limits ROS accumulation in a model of colon cancer [274]. Depletion of FAP^+^ cells improves the metabolism and functions of CD8^+^ T cells within tumours [275,276]. Conversely, the deregulated metabolism pathways can influence the expression and/or activity of ectoproteases. Hexokinase 2 (HK2) converts glucose to glucose-6-phosphate, the first committed step in glucose metabolism. HK2 contributes to ovarian cancer metastasis via a signalling pathway activating MMP9 expression [277]. Similarly, HK2 promotes the metastasis of colon cancer cells in nude mice through MMP2/9 upregulation [278]. Glucose transporter 1 (GLUT1), the main factor of the Warburg effect, is associated with poor prognosis in many tumours. GLUT1 enhances MMP2 expression and promotes the proliferation and migration of lung cancer cells [279]. Glutamine and pyruvate kinase (a key enzyme in the process of glycolysis) regulate migration and the invasion of ovarian cancer cells through the activation of MMP2/9 [280,281]. Elevated levels of 12-lipoxygenase (12-LOX) are associated with carcinoma progression and invasion. Overexpression of 12-LOX in prostate cancer cells results in the elevated expression of MMP9 [282]. By disrupting the cysteine–zinc binding, ROS stimulate the enzymatic activity of proMMP2/9 [283,284]. Indirectly, ROS mediate the increase in ADAM17 activity through the activation of the p38 signalling pathway in myeloid cells [285]. As a whole, these data highlight the link which exists between cancer-associated ectoproteases and cancer metabolism. Deregulation of metabolic enzymes and their metabolites in TME can increase the expression and/or function of cancer-associated ectoproteases. Conversely, ectoproteases can participate in the metabolic reprogramming in TME. Both ectoproteases and altered metabolism participate in tumour progression. Ongoing pharmacological approaches aim to exploit glycolytic enzymes in cancer therapy [267,268,286]. Thus, ectoproteases may serve as complementary therapeutic targets when aiming to influence metabolic pathways in cancer.

## 9. Concluding Remarks and Future Directions

Some of the ectoproteases discussed here are already useful as biomarkers in the identification of various solid and haematological cancers. For instance, APN is of diagnostic and/or prognostic relevance for patients with pancreatic, colon, and non-small cell lung cancer [5,6,9]. DPP4 is reported as a positive prognostic factor in ovarian cancer [287] and an established marker for diagnosis in cutaneous T cell lymphoma [288]. For the short term, it can be expected that FAP specific imaging will contribute to a better patient stratification and follow-up of therapy in several solid tumours [14,289]. We want to emphasize the importance of these ectoproteases in TME cell populations (including CSCs, differentiated cancer cells, CAFs, etc.) and tumour associated EVs that are promising not only as biomarkers, but also as therapeutic targets for cancer therapy. The list of ectoproteases as potential targets for cancer therapy is further expanding, as has already been seen with other ADAMs (including ADAM8, ADAM10, ADAM28), MT1-MMP/MMP14, the complex urokinase plasminogen (uPA)/uPAR, and neutral endopeptidase N/CD10 overexpressed in solid and haematological tumours [19,101,290,291]. Biomarker-guided trial design is recognized as pivotal in advancing the field of personalized medicine [292,293]. One of the best examples is breast cancer, where patients with abnormally high levels of HER2 protein in their tumour (HER2^+^ breast cancer) greatly benefit from combining trastuzumab (Herceptin^®^, anti-HER2) with chemotherapy [294]. Ectoproteases may also aid in identifying and developing novel strategies for cancer treatment, including the difficult-to-treat triple-negative breast cancer through the inhibition of ADAM17 or MMP9 [295,296]. This implies the use of validated ectoprotease inhibitors.

**Figure 3:**
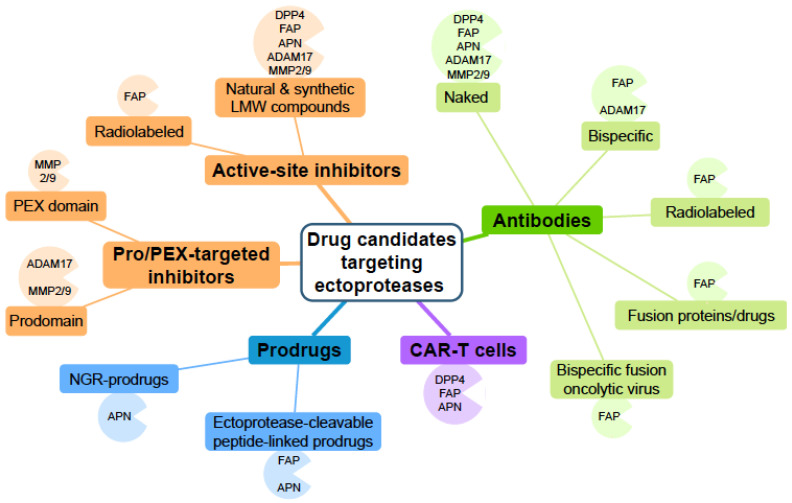
Schematic diagram summarizing the panel of drug candidates targeting cancer-associated ectoproteases in the context of cancer therapy.

So far, a large panel of biologicals and low molecular weight synthetic and natural compounds targeting these enzymes have been developed and evaluated (pre)clinically, as summarized in Figure 3. However, none of them are yet included in the standard of care treatments of malignancies. Among the number of drugs tested in clinical trials of solid or haematological tumours (Table 1), only the prodrug Melflufen (activated at the tumour site by APN) has successfully reached clinical trial phase III in relapsed refractory MM, which raises hope for this agent. The therapeutic potential of active-site inhibitors of APN, ADAM17, and MMP2/9 is currently limited, in part due to their lack of appropriate selectivity. Notwithstanding the ongoing development of more selective and metabolically stable molecules, targeting the catalytic activity of these ectoproteases remains, however, a challenge. Recent research efforts have opened up this field by considering ectoproteases in tumour-targeting strategies such as ectoprotease-targeted prodrugs, where one exploits the catalytic activity of the ectoprotease or ectoprotease-targeted radiotherapy using high-affinity small molecules, small molecules blocking exosites of ADAM17, and MMP2/9, NGR-prodrugs for APN, or mAbs (Table 1) (Figure 3). More advanced ectoprotease-directed mAb-based therapies include bispecific mAb-fusion proteins and costimulatory mAb-ligand fusion proteins/oncolytic virus (Table 1) (Figure 3). Similar to the mode of action of bispecific mAbs that crosslink tumour cells with T cells, CAR-T cells offer a novel strategy to treat cancer. FAP-targeting CAR-T cells of 2nd and 4th generation were recently tested in phase I trials of solid tumours (Table 1) (Figure 3). Still, the real added value and broad applicability of these innovative therapies need to be confirmed in the future.

Even more recently, siRNA therapeutics paved their way to the clinic [297,298]. The FDA’s approval of Patisiran for the treatment of hereditary transthyretin amyloidosis is the best example of the potential of siRNA therapeutics for treating various diseases [297,298]. siRNA molecules are used to block the expression of genes involved in cancer. Current efforts are being made on combinations of siRNA and chemotherapeutic drug delivery systems for the treatment of multidrug resistant cancers [297]. Numerous studies have previously indicated that the above ectoproteases can be “manipulated” by siRNA, resulting in both the in vitro and in vivo inhibition of tumour cell growth and migration, enhanced sensitivity to chemotherapeutic agents, and enhanced survival of mouse xenograft models of cancer [299,300,301,302,303,304,305,306,307,308]. Whether this siRNA strategy opens a new avenue for achieving a “knock-out” of cancer-associated ectoproteases is not yet a clinical reality, but deserves attention.

Last, but not least, the importance of metabolic reprogramming in cancer biology is being unveiled. Indeed, metabolic changes in cancer cells represent a novel opportunity for combination therapy approaches [286]. One important new hallmark of cancer-associated ectoproteases concerns their participation in tumour metabolic reprogramming and vice versa. Cotargeting ectoproteases and glycolytic enzymes may offer new therapeutic options to kill cancer cells.

To conclude, highly selective drugs targeting cancer-associated ectoproteases are currently in preclinical and clinical evaluation, and illustrate that this field of research is exciting and promising. Integrating the scientific progress and the challenges discussed in this Review may further stimulate research in the field of these and other ectoenzymes as promising pharmaceutical targets in a combined, personalized approach towards tumour elimination.

## Figures and Tables

**Table 1:** Selected results of terminated, completed, ongoing, or recruiting clinical trials with drugs targeting cancer-associated ectoproteases.

Drug	Class	Main Effect	Disease Setting	Phase	Study	Refs.
DPP4/CD26						
Linagliptin	Small molecule	Inhibiting DPP4 enzymatic activity thereby protecting chemokine agonist activity	Oesophagogastric tumours	I/II	NCT03281369 EUDRACT 2016-004529-17 (Recruiting)	
			Non-small cell lung cancer	I/II	NCT03337698 EUDRACT 2017-001267-21 (Recruiting)	
Sitagliptin	Small molecule	Inhibiting DPP4 enzymatic activity thereby protecting chemokine agonist activity	Hepatocellular carcinoma	I	NCT02650427 EUDRACT 2015-002968-17 (Completed)	[56]
		Accelerating engraftment in adults receiving umbilical cord blood transplantation	Haematological malignancies	I/II	NCT01720264 (Completed)	[66,67,68]
YS110	Antibody	Promoting internalization and nuclear accumulation of DPP4 leading to cell growth inhibition	Advanced malignant pleural mesothelioma	I/II	NCT03177668 (Completed)	[67,68]
**FAP/Seprase**						
^177^Lu-FAP-2286	Peptido- mimetic (theranostic)	Eliminating FAP^+^ CAFs and bystander tumour cells	Solid tumours	I/II	LuMIERE NCT04939610 (Recruiting)	[78]
RG7827/RO7122290	Bispecific antibody- fusion protein	Crosslinking FAP^+^ cells with 4-1BB^+^ T cells leading to T and NK cell activation	Metastatic colorectal tumour	Ib/II	NCT04826003 (Recruiting)	[79,80]
			Advanced solid tumours	Ia/Ib	EUDRACT 2017-003961-83 (Completed)	
			Urothelial carcinoma	Ib/II	EUDRACT 2017-004634-28 (Ongoing)	
RO7300490	Bispecific antibody- fusion protein	Crosslinking FAP^+^ cells with CD40^+^ APCs leading to APC immune response	Solid tumours	I	NCT04857138 EUDRACT 2020-004489-21 (Recruiting)	
RG7461/RO6874281/ Simlukafusp α	Bispecific antibody- fusion protein	Crosslinking FAP^+^ cells with IL2R^+^ T cells, leading to T cell immune response	Metastatic/advanced renal cell carcinoma	I	NCT03063762 EUDRACT 2016-003528-22 (Completed)	[81]
			Advanced solid tumours (breast, head, and neck)	I	NCT02627274 EUDRACT 2015-002251-97 (Completed)	[79]
			Recurrent/metastatic cervical squamous cell tumours	II	NCT03386721 EUDRACT 2017-003182-94 (Completed)	[82]
			Metastatic melanoma	I	NCT03875079 EUDRACT 2018-003872-11 (Active, not recruiting)	
			Pancreatic tumours	Ib/II	NCT03193190 EUDRACT 2016-004126-42 (Active, not recruiting)	
Anti-FAP CAR-T cells	CAR-T cells (2nd generation)	Eliminating tumour FAP^+^ CAFs	Malignant pleural mesothelioma	I	NCT01722149 (Completed)	[83,84]
Autologous anti-nectin4/anti-FAP CAR-T cells	CAR-T cells (4th generation)	Eliminating tumour Nectin4^+^ cells and FAP^+^ CAFs	Nectin4^+^ advanced malignant solid tumours	I	NCT03932565 (Recruiting)	
NG-641	Bispecific antibody- fusion oncolytic virus	Eliminating virus-infected tumour cells, crosslinking FAP^+^ cells with CD3^+^ T cells leading to T cell immune response	Metastatic/ advanced epithelial tumours	I	STAR NCT04053283 (Recruiting)	
**APN/CD13**						
CHR2797/ Tosedostat	Small molecule	Inhibiting APN enzymatic activity	Advanced pancreatic ductal adenocarcinoma	Ib/II	NCT02352831 (Terminated)	[85]
			Myelodysplastic syndromes	II	NCT02452346 (Completed)	[86]
NGR-TNFα	Peptide-based drug	Eliminating tumour-associated vasculature	Primary central nervous system lymphoma	II	NCT03536039 (Recruiting)	[87]
			Metastatic/advanced small cell lung cancer	II	NCT00483509 (Completed)	[88]
			Malignant pleural mesothelioma	III	NCT01098266 (Completed)	[89]
NGR-tTF	Peptide-based drug	Eliminating tumour-associated vasculature	Recurrent/refractory tumours (sarcoma, melanoma, lung, colon, liver, thyroid, lymphoma)	I	NCT02902237 (Completed)	[90]
J1/Melflufen	Peptide–drug conjugate	Delivering alkylating melphalan to tumour cells, leading to tumour cell elimination	Relapsed refractory multiple myeloma	I/II	HORIZON NCT02963493 (Completed) ANCHOR NCT03481556 (Completed)	[91,92]
				III	OCEAN NCT03151811 (Completed)	[92,93]
**ADAM17/TACE**						
INCB7839/ Aderbasib	Small molecule	Blocking HB-EGF shedding and EGFR/EGF ligand signalling	Diffuse large B-cell non-Hodgkin lymphoma	I/II	NCT02141451 (Completed)	[94]
**MMP9**						
GS-5745/ Andecaliximab	Allosteric antibody	Inhibiting MMP9 activation and MMP9 activity by binding to (pro)MMP9	Advanced solid tumours (pancreatic, non-small cell lung, colorectal, oesophagogastric, breast)	I	NCT01803282 (Completed)	[95,96]
			Oesophagogastric tumours	I/Ib	NCT02862535 (Terminated)	[97]
				III	NCT02545504 2015-001526-42 (Completed)	[98]

Clinical trial identifiers from Clinicaltrials.gov (accessed on 19 December 2021): NCTxxxxxxxx; EU Clinical Trials Register: 20xx-00xxxx-xx.

## Supplementary Materials

The following supporting information can be downloaded at: https://www.mdpi.com/article/10.3390/cancers14030624/s1, Table S1: FAP-targeted diagnostic tracers for tumour imaging.
