# Supplementary material for: Validating Cell Surface Proteases as Drug Targets for Cancer Therapy: What Do We Know, and Where Do We Go?
Source: Cancers (Basel). 2022 Jan 26;14(3):624. doi: 10.3390/cancers14030624 (PMC8833564; doi:10.3390/cancers14030624)
Supplement: Supplementary file 1 [file cancers-14-00624-s001.zip › cancers-1541238-supplementary.pdf]

cancers MDPI

Table S1. FAP-targeted diagnostic tracers for tumour imaging.

Tracer	Disease Setting	Phase	Study	Refs.
^{68}Ga-DOTA- FAP-2286	Solid tumours	I	NCT04621435 (Recruiting)	
^{68}Ga-DOTA-FAPI-04	Malignant Neoplasm	NA	NCT04554719 (Recruiting)	[1–3]
	Malignant Neoplasm	NA	NCT05034146 (Recruiting)	
	Solid tumours	NA	ChiCTR2100044131 (Recruiting)	[4–6]
	Oral Carcinoma	I	NCT05003427 (Recruiting)	
	Various types of cancer	I	NCT04499365 (Recruiting)	[7]
	Epithelial Ovarian Cancer	II	NCT04504110 (Recruiting)	
	Lymphoma	NA	ChiCTR2000032437; NCT04367948 (Not yet recruiting)	[8]
	Solid tumours	NA	ChiCTR2100045757 (Recruiting)	[9]
Al[^{18}F]-NOTA-FAPI-04	Malignant solid tumours	NA	ChiCTR2100051406 (Not yet recruiting)	
	Tumours	NA	ChiCTR2100048334 (Recruiting)	
	Lymphoma	NA	ChiCTR2000032437; NCT04367948 (Not yet recruiting)	[8]
	Solid tumours	NA	ChiCTR2000038080 (Recruiting)	[10]
Al[^{18}F]-NOTA-FAPI-42	Solid tumours	NA	ChiCTR2100045757 (Recruiting)	[9]
^{68}Ga-DOTA- FAPI-46	(Metastatic) Sarcoma	I	NCT04457258 (Recruiting)	
	Breast, Colon, Esophageal, Gastric, Head and Neck, Lung, Kidney, Ovarian, Pancreatic, Renal and Uterus Cancer	I	NCT04147494 (Recruiting)	[11,12]
	Brain, Bladder, Urothelial, Testicular, Skin, Thyroid, Hepatocellular, Thymus, Pleural, Cervical, Adrenal and Hematologic cancer and Cholangiocarcinoma and Neuroendocrine tumours	I	NCT04459273 (Recruiting)	
	Prostate carcinoma	I	NCT04457232 (Recruiting)	
	Different FAP^{+} cancer types	II	2021-000148-23 (Ongoing)	
^{68}Ga-DOTA-FAPI (specific inhibitor unknown)	Oral Squamous Cell Carcinoma	NA	NCT05030597; ChiCTR2100050144 (Not yet recruiting)	

FAP-targeted diagnostic tracers for tumour imaging, based on small molecule FAP-inhibitors linked to a chelator-captured isotope. Clinical trial identifiers from Clinicaltrials.gov: NCTxxxxxxxx; EU Clinical Trials Register: 20xx-00xxxx-xx; Chinese Clinical Trial Register: ChiCTR2x000xxxxx.

References

1. Zhang, X.; Song, W.; Qin, C.; Liu, F.; Lan, X. Non-malignant findings of focal 68Ga-FAPI-04 uptake in pancreas. *Eur. J. Pediatr.* **2021**, *48*, 2635–2641, https://doi.org/10.1007/s00259-021-05194-6.
2. Qin, C.; Liu, F.; Huang, J.; Ruan, W.; Liu, Q.; Gai, Y.; Hu, F.; Jiang, D.; Hu, Y.; Yang, K.; et al. A head-to-head comparison of 68Ga-DOTA-FAPI-04 and 18F-FDG PET/MR in patients with nasopharyngeal carcinoma: a prospective study. *Eur. J. Pediatr.* **2021**, *48*, 3228–3237, https://doi.org/10.1007/s00259-021-05255-w.
3. Qin, C.; Song, Y.; Liu, X.; Gai, Y.; Liu, Q.; Ruan, W.; Liu, F.; Hu, F.; Lan, X. Increased uptake of 68Ga-DOTA-FAPI-04 in bones and joints: metastases and beyond. *Eur. J. Nucl. Med. Mol. Imaging* **2021**, 1–12, https://doi.org/10.1007/s00259-021-05472-3.
4. Liu, H.; Yang, X.; Liu, L.; Lei, L.; Chen, Y. Incidental Detection of Sinonasal Inverted Papilloma With 68Ga-FAPI PET/CT in a Pa-tient With Esophageal Cancer. *Clinical Nuclear Medicine* **2021**, *46*, 845-846.
5. Yang, X.; Liu, H.; You, Z.; Gong, W.; Chen, Y. Increased 68Ga-FAPI Uptake in Intramuscular Gluteal Hematoma in a Patient With Hemophagocytic Syndrome. *Clin. Nucl. Med.* **2021**, *46*, 1022–1023, https://doi.org/10.1097/rlu.0000000000003771.
6. Lan, L.; Liu, H.; Wang, Y.; Deng, J.; Peng, D.; Feng, Y.; Wang, L.; Chen, Y.; Qiu, L. The potential utility of [68 Ga]Ga-DOTA-FAPI-04 as a novel broad-spectrum oncological and non-oncological imaging agent—comparison with [18F]FDG. *Eur. J. Nucl. Med. Mol. Imaging* **2021**, 1–17, https://doi.org/10.1007/s00259-021-05522-w.
7. Zheng, S.; Lin, R.; Chen, S.; Zheng, J.; Lin, Z.; Zhang, Y.; Xue, Q.; Chen, Y.; Zhang, J.; Lin, K.; et al. Characterization of the benign lesions with increased 68Ga-FAPI-04 uptake in PET/CT. *Ann. Nucl. Med.* **2021**, *35*, 1312–1320, https://doi.org/10.1007/s12149-021-01673-w.
8. Wang, G.; Jin, X.; Zhu, H.; Wang, S.; Ding, J.; Zhang, Y.; Yang, Z.; Wang, X. 68Ga-NOTA-FAPI-04 PET/CT in a patient with primary gastric dif-fuse large B cell lymphoma: comparisons with [18F] FDG PET/CT. *Eur. J. Nucl. Med. Mol. Imaging* **2021**, *48*, 647-8.
9. Hu, K.; Wang, L.; Wu, H.; Huang, S.; Tian, Y.; Wang, Q.; Xiao, C.; Han, Y.; Tang, G. [^{18}F]FAPI-42 PET imaging in cancer patients: optimal acquisition time, biodistribution, and comparison with [^{68}Ga]Ga-FAPI-04. *Eur. J. Nucl. Med. Mol. Imaging* **2021,** https://doi.org/ 10.1007/s00259-021-05646-z.
10. Wang, S.; Zhou, X.; Xu, X.; Ding, J.; Liu, S.; Hou, X.; Li, N.; Zhu, H.; Yang Z. Clinical translational evaluation of Al18F-NOTA-FAPI for fibroblast acti-vation protein-targeted tumour imaging. *Eur. J. Nucl. Med. Mol. Imaging* **2021**, *48*, 4259-4271.
11. Sonni, I.; Lee-Felker, S.; Memarzadeh, S.; Quinn, M.M.; Mona, C.E.; Lückerath, K.; Czernin, J.; Calais, J. 68Ga-FAPi-46 diffuse bilateral breast uptake in a patient with cervical cancer after hormonal stimulation. *Eur. J. Nucl. Med. Mol. Imaging* **2021**, *48*, 924–926, https://doi.org/10.1007/s00259-020-04947-z.
12. Mona, C.; Benz, M.; Hikmat, F.; Grogan, T.; Lueckerath, K.; Razmaria, A.A.; Riahi, R.; Slavik, R.; Grigis, M.; Carlucci, G.; et al. Validation of FAPi PET biodistribution by im-munohistochemistry in patients with solid cancers: A prospective exploratory study. *J. Clin. Oncol.* **2021**, 39, *15*, 3066.
